# Recombinant yeast VDAC2: a comparison of electrophysiological features with the native form

**DOI:** 10.1002/2211-5463.12574

**Published:** 2019-06-17

**Authors:** Andrea Magrì, Andonis Karachitos, Maria Carmela Di Rosa, Simona Reina, Stefano Conti Nibali, Angela Messina, Hanna Kmita, Vito De Pinto

**Affiliations:** ^1^ Department of Biomedical and Biotechnological Sciences University of Catania Italy; ^2^ Department of Biological, Geological and Environmental Sciences Section of Molecular Biology University of Catania Italy; ^3^ Department of Bioenergetics Faculty of Biology Institute of Molecular Biology and Biotechnology Adam Mickiewicz University Poznan Poland; ^4^ National Institute for Biomembranes and Biosystems Section of Catania Italy

**Keywords:** electrophysiology, heterologous expression, mitochondria, mitochondrial porins, planar lipid bilayer, VDAC2, yeast, yeastVDAC2, yVDAC2

## Abstract

Voltage‐dependent anion channel isoform 2 of the yeast *Saccharomyces cerevisiae* (yVDAC2) was believed for many years to be devoid of channel activity. Recently, we isolated yVDAC2 and showed that it exhibits channel‐forming activity in the planar lipid bilayer system when in its so‐called native form. Here, we describe an alternative strategy for yVDAC2 isolation, through heterologous expression in bacteria and refolding *in vitro*. Recombinant yVDAC2, like its native form, is able to form voltage‐dependent channels. However, some differences between native and recombinant yVDAC2 emerged in terms of voltage dependence and ion selectivity, suggesting that, in this specific case, the recombinant protein might be depleted of post‐translational modification(s) that occur in eukaryotic cells.

**Abbreviations**
LDAO: lauryldimethylamine oxide
MOM: mitochondrial outer membrane
PLB: planar lipid bilayer
PTM: post‐translational modification
ROS: reactive oxygen species
VDAC: voltage‐dependent anion channel
yVDAC2: yeast voltage‐dependent anion channel isoform 2

The voltage‐dependent anion channel (VDAC) proteins, also known as mitochondrial porins, are the most abundant proteins in the mitochondrial outer membrane (MOM) of all eukaryotes. Characterized by a molecular mass of 30–32 kDa, VDAC proteins allow for the continuous exchange of metabolites (ATP/ADP, NAD^+^/NADH, Krebs cycle intermediates) and ions (Na^+^, Cl^−^, Mg^2+^, Ca^2+^) between mitochondria and cytosol. Therefore, they play a crucial role in mitochondrial bioenergetics 1, 2, 3. In mammals, where three different but conserved isoforms are expressed (VDAC1, VDAC2 and VDAC3) 4, evolution has conferred to each isoform peculiar additional functions beyond the metabolic role. For example, VDAC1 and VDAC2 participate differently in the regulation of cell death 5, 6, 7, 8, while recent literature suggests the involvement of VDAC3 in reactive oxygen species (ROS) homeostasis 9, 10. The three‐dimensional structure of mammalian VDAC1 has been shown to involve a transmembrane β‐barrel structure, formed by 19 amphipathic, mostly anti‐parallel β‐strands, with the exception of the N‐terminal domain, which is structured as an α‐helix and partially exposed to the cytosol 11, 12, 13. A similar result was obtained for zebrafish VDAC2 14 and for other species by homology modeling studies 15, suggesting a conserved structure of VDAC across evolution.

Differently from mammals, the budding yeast *Saccharomyces cerevisiae* has only two distinct VDAC genes, *POR1* and *POR2*, encoding two different VDAC isoforms. However, only yVDAC1 is widely considered essential for mitochondrial metabolism. Indeed, the inactivation of the *POR1* gene, as in the Δ*por1* mutant strain, resulted in a strong inhibition of cell growth in non‐fermentable conditions, especially at the restrictive temperature of 37 °C 16. On the contrary, the inactivation of the *POR2* gene resulted in an undetectable phenotype, since Δ*por2* cells were able to grow in a comparable way to the wild‐type 16. Overall, the channel‐forming ability of yeast voltage‐dependent anion channel isoform 2 (yVDAC2) has been questioned for many years. Contrasting results were indeed obtained upon *POR2* overexpression; namely, a complete recovery of Δ*por1* yeast growth on glycerol was detected 16, 17, suggesting a channel activity for yVDAC2, at least under particular conditions. Only recently, an electrophysiological analysis has clearly shown that yVDAC2 is able to form channels in artificial membranes, with features resembling many other members of the VDAC family 18.

In a previous work, we purified yVDAC2 directly from mitochondria extracted from Δ*por1* cells overexpressing *POR2*, under native conditions 18. In parallel, the same protein was prepared by heterologous expression in a bacterial system, purified and refolded as previously performed for many other VDAC isoforms and mutants 19, 20, 21, 22. In this work, we analyzed the electrophysiological features of the recombinant yVDAC2. Our results indicate that the protein is able to form channels in artificial membranes, with similar features to native one but, at the same time, with differences in terms of voltage sensitivity and ion selectivity.

## Materials and methods

### Cloning of yVDAC2

The sequence encoding yVDAC2 was obtained by reverse transcription from the total RNA previously extracted from *S. cerevisiae* BY4742 (EUROSCARF, Frankfurt, Germany). Reverse transcription was performed by using the AffinityScript Multi‐Temp RT and RT‐PCR system (Agilent, Santa Clara, CA, USA) according to the manufacturer's protocol. The corresponding cDNA was amplified by PCR using the following primers: forward 5′‐TTTTGCTAGCATGGCACTACGATTTTTCAACGAT‐3′ and reverse 5′‐TTTTCTCGAGGGGCGAGAACGATAGAGACCA‐3′. The yVDAC2 cDNA was cloned in the bacterial expression vector pET‐21b (Novagen, Madison, WI, USA) by NheI/XhoI digestion, in frame with the His‐tag at the C‐terminal part. The construct was verified by sequencing.

### Expression, purification and refolding of yVDAC2 protein

*Escherichia coli* BL21 (DE3) cells were transformed with pET‐21b plasmid containing the yVDAC2 sequence. Transformed cells were grown to an optical density (λ = 600 nm) of 0.6, and the protein expression was induced by addition of 0.4 mm isopropyl‐β‐d‐thiogalactopyranoside (IPTG) (Sigma‐Aldrich, Saint Louis, MO, USA), for 3 h at 30 °C. Cells were harvested by centrifugation and lysed in buffer B (8 m urea, 1 mm NaH_2_PO_4_, 0.01 mm Tris/HCl, pH 8.0) overnight at 4 °C. The total protein lysate was clarified by centrifugation and loaded onto Ni‐NTA agarose (Thermo Fisher Scientific, Waltham, MA, USA) packed column, previously equilibrated with buffer B. The column was then washed twice with five volumes of buffer C (8 m urea, phosphate buffer, pH 6.3), and proteins were purified by elution with five volumes of buffer E (8 m urea, phosphate buffer, pH 3.5). The eluted protein was refolded by incubation at 4 °C in a 10‐fold volume of refolding buffer [25 mm Tris, 100 mm NaCl, 1 mm EDTA, 1% (v/v) lauryldimethylamine oxide (LDAO, Sigma‐Aldrich), pH 7.0]. Then, the protein solution was dialyzed against 100 volumes of a dialysis buffer (25 mm Tris, 1 mm EDTA, 0.1% LDAO, pH 7.0) with Thermo Scientific Slide‐A‐Lyzer Dialysis Cassettes (Thermo Fisher Scientific) (3.5 K MWCO). The solution containing the refolded proteins was clarified by centrifugation (60 000 ***g***, 1 h) and loaded into a size exclusion chromatography column (Superdex 200 Increase 30/100, GE Healthcare, Chicago, IL, USA) in SEC buffer (150 mm NaCl, 20 mm Tris/HCl, pH 8.0, 1 mm DTT 0.1% LDAO) to separate aggregates from monomers. Elution fractions, corresponding exclusively to the monomer peak, were collected.

### Electrophysiological analysis of recombinant yVDAC2

The refolded yVDAC2 was reconstituted into a planar lipid bilayer (PLB) system as previously described in 20, 21, 22. Bilayers of approximately 80–100 pF capacity made of asolectin from a soybean phospholipid mixture (Sigma‐Aldrich) at a concentration of 20 mg·mL^−1^ in *n*‐decane were used. Channel insertion was obtained by addition of 0.5–5 μL of protein solution to the *cis* side of the cuvette containing approximately 3 mL of aqueous solution (1 m KCl, 10 mm Hepes, pH 7.0). Data were acquired using a Bilayer Clamp amplifier (Warner Instruments, Hamden, CT, USA) at 100 μs per point, filtered at 300 Hz and analyzed offline using the pclamp program set (version 10; Molecular Devices, San Jose, CA, USA). Channel conductance (*G*) was calculated from current (*I*) measurements in the presence of an applied constant voltage (*V*_m_) of +10 mV, as the *I*/*V*_m_ ratio. Distribution of conductance was obtained from six independent reconstitution experiments, each showing several channel insertions.

### Voltage dependence analysis of yVDAC proteins

Analysis of voltage dependence of recombinant yVDAC2 was performed in 1 m KCl, 10 mm Hepes, pH 7.0, by application of two distinct voltage ramps including positive and negative potentials (amplitude ±90 mV, time 100 s). At least three independent multi‐channel experiments were performed. Values of *G* and *G*_max_, indicating respectively the conductance value at a given *V*_m_ and the maximal conductance, were calculated for the recombinant yVDAC2. Control experiments were performed using the native yVDAC1 and yVDAC2 produced as previously described 18. The relative conductance was calculated as the *G*/*G*_max_ ratio and plotted as a function of the voltage using prism 7 software (GraphPad Software Inc., La Jolla, CA, USA). Three independent experiments were performed for each protein and data are shown as the mean ± SEM.

### Ion selectivity measurement of yVDAC2

Selectivity measurements were performed for the recombinant yVDAC2 in a 0.1 m/1 m *cis*/*trans* gradient of KCl in a voltage range of ±90 mV, by increasing *V*_m_ with discrete steps of ±5 mV for 45 s. Values of conductance were plotted as a function of *V*_m_, and linear regression was applied with prism 7 software. The permeability ratio of cation K^+^ (*P*_C_) over anion Cl^−^ (*P*_A_) was calculated from the reversal potential (*V*_r_) using the Goldman–Hodgkin–Katz equation, according to 23. Six independent selectivity measurements were performed with both a single‐ and a multi‐channel approach.

## Results

### Reconstituted recombinant yVDAC2 forms channels of conductance similar to the native protein

**Figure 1:**
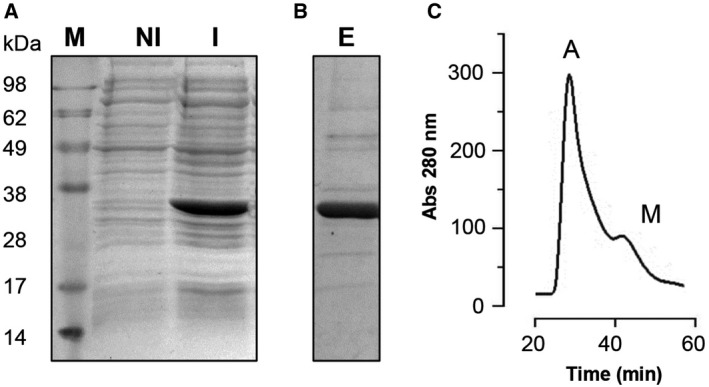
Expression and purification of yVDAC2 in bacterial system. (A) SDS/PAGE analysis of whole *Escherichia coli* BL21 (DE3) lysate from cells transformed with the pET‐21b‐yVDAC2 construct, obtained in the presence of 0.4 m IPTG (I, induced). As a control, a sample that was not induced (NI) was used. Addition of IPTG allowed the expression of recombinant yVDAC2 as demonstrated by the strong band of the expected molecular mass (28–32 kDa) exclusively found in the induced sample. ‘M’ indicates the molecular mass marker. (B) SDS/PAGE of eluate (E) obtained after Ni‐NTA chromatography of the total induced lysate. As shown, a predominant band of the expected molecular mass was detected. (C) Elution profile obtained by size‐exclusion chromatography of refolded protein. ‘A’ indicates the peak corresponding to aggregated proteins; ‘M’ indicates the peak corresponding to the monomeric protein. Only fractions corresponding to monomeric protein were collected and used going forward.

To analyze the electrophysiological properties of recombinant yVDAC2, the corresponding encoding sequence was introduced into *E. coli* BL21 (DE3) cells and the protein expression was achieved by IPTG induction, as verified by SDS/PAGE (Fig. 1A). The recombinant protein was extracted directly from inclusion bodies and purified by nickel affinity chromatography, exploiting the 6×His tag (Fig. 1B). Since the heterologous expression of membrane proteins in bacterial systems leads to loss of proper conformation and activity, the purified yVDAC2 was refolded and dialyzed. In order to avoid the presence of aggregates in the final protein solution, size exclusion chromatography was performed and the elution fractions, corresponding to the monomers (Fig. 1C), were collected and used in our study. Finally, the electrophysiological features of recombinant yVDAC2 were analyzed at the PLB.

**Figure 2:**
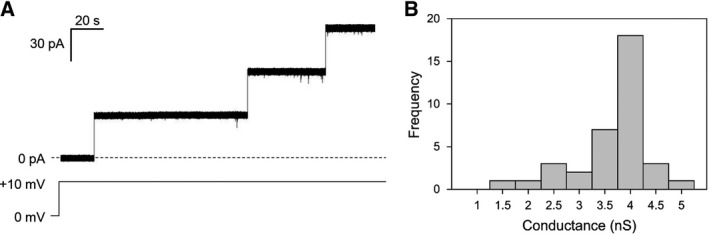
Pore‐forming activity analysis of recombinant yVDAC2 in artificial membranes. (A) Representative trace of yVDAC2 activity recorded at the PLB. Channels insertion was achieved after addition of 0.5–5 μL of protein solution into the *cis* side of the cuvette, in presence of a lipid bilayer. Discrete steps indicate single channel insertion. Experiment was performed in 1 m KCl, 10 mm Hepes, pH 7, at a constant applied voltage of +10 mV. (B) Distribution of conductance values obtained for *n* = 36 channels inserted in the membrane. Most of the channels showed conductance values between 3.5 and 4 nS, resulting in an average conductance value of 3.76 ± 0.94 nS (SD, *n* = 36).

The representative current trace displayed in Fig. 2A clearly shows that recombinant yVDAC2 was able to form channels in artificial membrane, since insertion of three different channels of similar size in a short time was obtained after the addition of the protein solution to the *cis* side of the cuvette. The histogram in Fig. 2B shows the distribution of conductance values calculated for *n* = 36 channels. The analysis, performed in 1 m KCl, 10 mm Hepes, pH 7, in the presence of an applied constant voltage of +10 mV, allowed for calculation of the average conductance, estimated as 3.76 ± 0.94 nS (SD, *n* = 36). This value perfectly overlaps our previous measurements showing an average conductance for the native yVDAC2 of about 3.6 nS in the same experimental conditions 18. Overall, our results indicate that, in artificial membrane, recombinant yVDAC2 forms channels of conductance similar to the native protein.

### Recombinant yVDAC2 is less sensitive to the applied voltage than the native protein

It is known that the conductance of VDAC proteins changes depending on the applied voltage. This feature, well conserved through evolution from yeast to human, is known as voltage dependence. In general, yeast or mammalian VDAC1 persists at low potentials in a stable highly conductive state, called the ‘open’ state. However, as the applied voltage rises (starting from ±20–30 mV), VDAC1 switches to several low‐conducting states, called ‘closed’ states 24, 25, 26, 27. We demonstrated previously that the native yVDAC2 shows a less pronounced voltage dependence in comparison to yVDAC1, because yVDAC2‐formed channel closure began from ±40–50 mV of applied voltage 18.

**Figure 3:**
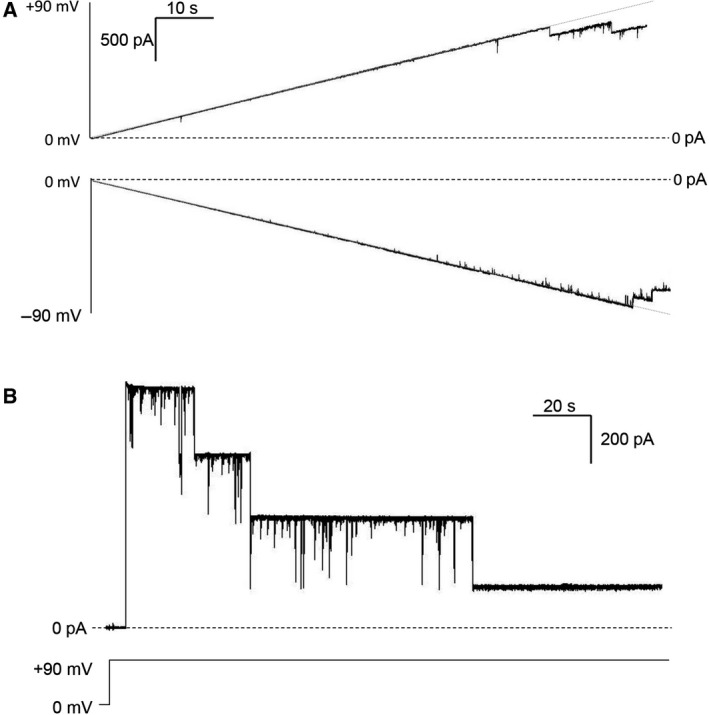
Voltage dependence of recombinant yVDAC2. (A) Representative traces of yVDAC2 activity at the PLB in presence of voltage ramps from 0 to +90 mV (upper trace) and from 0 to −90 mV (lower trace). Traces show that no change in the conductance was obtained at low positive or negative potentials. A significant reduction of channel conductance was obtained starting from ±80 mV. Experiments were performed in 1 m KCl, 10 mm Hepes, pH 7. (B) Representative trace of recombinant yVDAC2 recorded at the constant voltage of +90 mV for a prolonged time; *n* = 3 different channels inserted in the membrane. It is clear that application of high voltage promotes a step‐by‐step closure of yVDAC2 channels. The experiment was performed in 1 m KCl, 10 mm Hepes, pH 7.

In order to analyze the effect of voltage on recombinant yVDAC2 conductance, PLB experiments were performed by the application of voltage ramps from 0 to ±90 mV. The curves, displayed in Fig. 3A, indicate that recombinant yVDAC2 is completely insensible to low positive and negative voltages, since no closure was detected either at potentials that normally close yeast or mammalian VDAC1 or at voltages able to close native yVDAC2. Only the application of high voltages, starting from ±70–80 mV, induced a channel closure, which occurred symmetrically at both positive and negative potentials, suggesting that recombinant yVDAC2 is significantly less voltage sensitive than the native protein. Furthermore, current across recombinant yVDAC2 was monitored upon application of the constant voltage +90 mV. As shown in the representative trace in Fig. 3B, a complete closure of the channels occurred only after a prolonged exposure to high potential, highlighting the recombinant yVDAC2 limited sensitivity to voltage.

**Figure 4:**
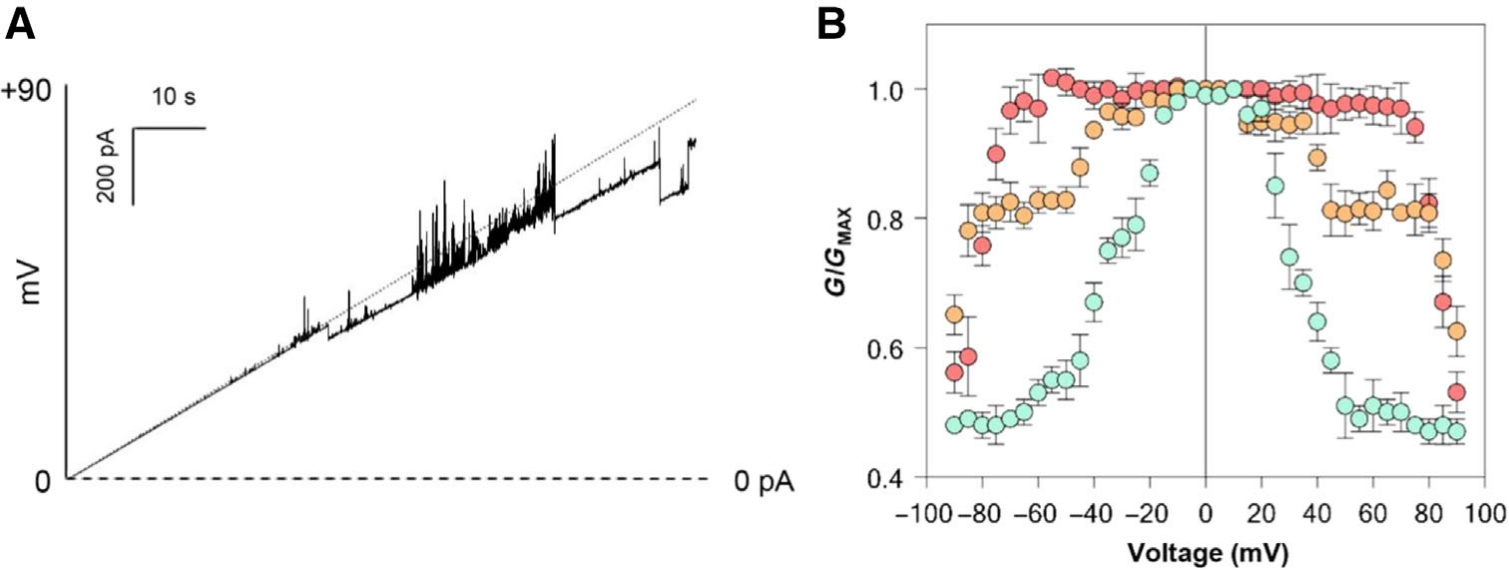
Comparative analysis of relative conductance for yVDAC proteins. (A) A representative trace of native yVDAC2 activity in presence of voltage ramps from 0 to +90 mV. The trace shows that the protein undergoes to partial closure at +40–50 mV and, as the voltage increases, channels become noisy. However, a stable reduction of channel conductance was obtained starting from ±80 mV. Experiments were performed in 1 m KCl, 10 mm Hepes, pH 7. (B) Analysis of relative conductance (*G*/*G*_max_) (±SEM) of *n* = 3 independent experiments showing the different voltage‐dependent features of yVDAC proteins. Pale green circles refer to yVDAC1, orange circles refer to native yVDAC2 results, and red circles refer to recombinant yVDAC2. As reported, both native and recombinant yVDAC2 remain in an open state up to application of high voltages, starting from ±80–90 mV, when they switch to the closed state. Experiments were performed in 1 m KCl, 10 mm Hepes, pH 7.

The electrophysiological behavior of the native protein was then analyzed in the presence of similar high voltages, by applying a voltage ramp from 0 to +90 mV. According to our previous data 18, native yVDAC2 began to close around +40–50 mV, as shown by the trace in Fig. 4A. However, the application of higher potentials (i.e. +80 mV) promoted a further channel closure, similarly to that previously shown by the recombinant protein (see Fig. 3A). To deeply analyze this aspect, the values of conductance (*G*) obtained at each applied voltage for both native and recombinant proteins, as well as for the native yVDAC1, were normalized relative to the maximal conductance (*G*_max_). Then, as shown in Fig. 4B, relative conductance values (*G*/*G*_max_) were plotted as a function of the applied voltage (amplitude of ±90 mV). As expected, yVDAC1 conductance (pale green circles) significantly decreased starting from ±20–30 mV, reaching a stable level of closure characterized by a decrease at about 50% of the maximal conductance. Native yVDAC2 (orange circles) began to close around ±40–50 mV and its conductance at these voltages showed a decrease of about 20%, in a similar manner to that shown previously 18. It is clear that this channel is not able to reach a channel closure similar to that observed for yVDAC1. However, the application of high voltages, up to ±80–90 mV, promoted a further 10–15% decrease of its relative conductance.

The results shown in Fig. 4B also confirmed the overall attenuation of voltage dependence in the case of recombinant yVDAC2 (red circles) observed in Fig. 3. Furthermore, the application of voltages up to ±60–70 mV did not promote any significant decrease in the channel conductance. Conversely, the application of potentials ±80–90 mV caused channel closure, as *G*/*G*_max_ values almost overlap those calculated for both native yVDAC1 and yVDAC2 at similar voltages. Although the recombinant yVDAC2 still shows voltage dependence, our results suggest a decrease of voltage sensitivity even with respect to the native form.

### Recombinant yVDAC2 displays exclusively cation‐selective states

**Figure 5:**
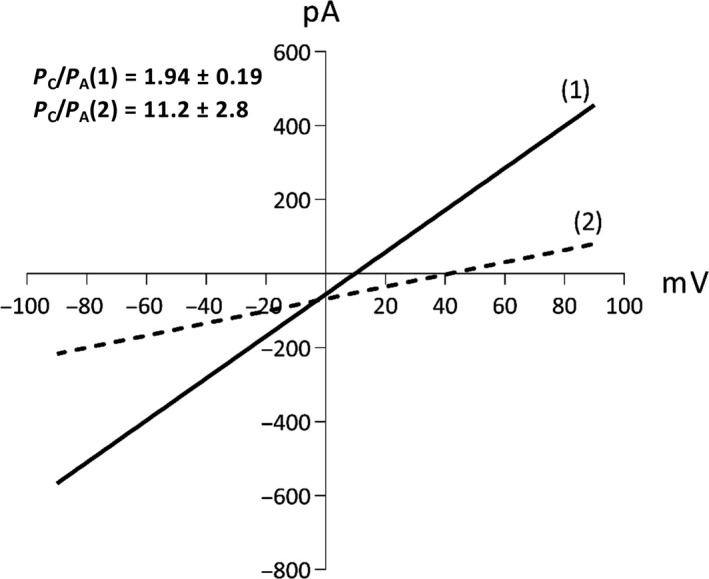
Ion selectivity of recombinant yVDAC2. Representative graph of current–voltage relationship of yVDAC2 recombinant protein performed in 10‐fold gradient of 1 m KCl obtained by application of voltage steps for a prolonged time. The conductance (slope) and the reversal potentials were calculated from the extrapolated regression lines of the different conducting states of the channel. The multi‐channel experiment shown was performed with *n* = 8 channels inserted in the membrane. Numbers indicate the different states: 1, cationic high‐conducting state; 2, cationic low‐conducting state.

It is known that VDAC displays an anionic selectivity in the high‐conducting ‘open’ state and a less anionic or more cationic selectivity in the low‐conducting ‘closed’ state 28, 29. In the previous work, the presence of 1 and 0.1 m KCl in the *cis* and *trans* side of the cuvette, respectively, allowed for detection of the presence of different selectivity states, three in total for native yVDAC2. Two of them appeared to be high‐conducting states but with an opposite selectivity, while the third was a very low‐conducting state characterized by an extremely pronounced cation selectivity 18. In order to analyze the ion selectivity of recombinant yVDAC2, current values, measured in a 10‐fold gradient of KCl and obtained upon application of discrete voltages in the range of ±90 mV, were plotted as a function of the applied voltage. Linear regressions of the obtained data (Fig. 5) indicated the presence of only two distinct states of recombinant yVDAC2, i.e. a main high‐conducting state (called state 1 in Fig. 5) and a low‐conducting state (state 2 in Fig. 5) and that both states displayed a cation selectivity.

The Goldman–Hodgkin–Katz equation was then used to calculate the ratio between permeability of cations over anions (*P*_C_/*P*_A_) for the two states of recombinant yVDAC2, using a reversal potential corresponding to the voltage at zero current 23. The *P*_C_/*P*_A_ value obtained for state 1 was 1.94 ± 0.19 and the value obtained for state 2 was 11.2 ± 2.8. For comparison, the corresponding *P*_C_/*P*_A_ values obtained for native yVDAC2 were 2.17 ± 0.11 and 16.24 ± 6, respectively 18. This clearly indicates that recombinant and native yVDAC2 are very similar in terms of cationic strength. In particular, the lowest conducting state determined for both variants of yVDAC2 is characterized by a pronounced selectivity for cations over anions, a feature found exclusively for yVDAC2. At the same time, a significant difference between native and recombinant protein emerged, since no anionic state was found for the recombinant one.

## Discussion

So far, the second VDAC isoform of the yeast *S. cerevisiae* has been considered to be one of the most elusive members of the VDAC family. Indeed, since the *POR2* gene discovery, the story of yVDAC2 has been controversial. Differently from *POR1*, the genetic inactivation of *POR2* has no consequence for yeast growth on non‐fermentable carbon sources 16. Furthermore, no data about yVDAC2 reconstitution in liposomes and/or artificial membranes were available for a long time, raising the suspicion (now proved wrong 18) that the yVDAC2 protein was completely devoid of channel function. On the contrary, the overexpression of the *POR2* gene in Δ*por1* cells promoted the restoration of the yeast wild‐type growth phenotype. This effect can be achieved when the *POR2* sequence is transcribed under the control of a strong promoter 16 or is activated by an external factor, such as the serendipitously found human SOD1, which stimulates *POR2* overexpression 17. These findings in turn suggest that the contribution of yVDAC2 to the permeability of the yeast MOM is a consequence of its expression level, providing a logical explanation for the recovery of Δ*por1* cell growth 16 and of the mitochondrial functionality 17. To definitively clarify the functional properties of yVDAC2, we have recently analyzed the electrophysiological features of this protein reconstituted into artificial membranes by using two parallel approaches. As shown in our previous work, we isolated yVDAC2 directly from Δ*por1* mitochondria, after yeast cell transformation with an expression plasmid carrying the corresponding yVDAC2 cDNA sequence. This strategy allowed us to increase *POR2* transcript concentration and consequently the corresponding protein amount, which simplified the protein isolation under the native condition 18. Alternatively, yVDAC2 was produced as a recombinant protein in *E. coli* cells, a procedure commonly used for large‐scale protein production, since it allows a high yield of protein to be obtained in a relatively short time with minimal cost and effort. The same approach was previously used by our group for the characterization of human VDAC3 20 and its cysteine mutants 21, and for the production of human VDAC1 suitable for interaction studies with proteins involved in neurodegenerative diseases 22, 30. Furthermore, an identical approach was used by Hiller and coauthors to obtain the human VDAC1 preparation for NMR studies aimed at identifying its three‐dimensional structure 11, 31 and by Abramson's group to purify the mouse VDAC1, later crystalized 13, confirming the strength of this method. With this perspective, the yVDAC2 cDNA was cloned into a specific expression vector and the protein was expressed in bacterial cells. Since membrane proteins localize into inclusion bodies, bacterial lysis occurred upon denaturing conditions, with the loss of the native conformation. Therefore, once isolated, in order to obtain a perfectly active protein, the recombinant protein must undergo a refolding procedure using a well‐established protocol 11, 19, 20, 21, 22.

Recombinant yVDAC2 was then characterized by reconstitution in a PLB system and previous data were used as a reference. Our reconstitution experiments clearly indicate the ability of the recombinant protein to form channels in artificial membranes, with an average conductance value identical to that previously found for the native protein. This result confirmed also the appropriateness of our refolding method. The analyses of voltage dependence indicate that the recombinant yVDAC2 is less sensitive to voltage than native yVDAC1 and yVDAC2, although the voltage dependence of the latter is also weaker than that of the former. Accordingly, only application of a high potential is able to promote a strong attenuation (approximately halving) of relative conductance of native yVDAC2, allowing the protein to reach a similar closure level to that displayed by yVDAC1. Similarly, the recombinant yVDAC2 undergoes a complete and stable closure exclusively at high potentials, such as ±80–90 mV.

Another significant difference among native and recombinant yVDAC2 concerns ion selectivity. In our previous work, we found that native yVDAC2 displays the anion‐selective state typical of most VDAC proteins, and two different cation‐selective states 18. Importantly, both cation‐selective states are observed for recombinant yVDAC2 as indicated by values of *P*_C_/*P*_A_, but no anion‐selective state was found for the recombinant protein. Although the presence of the highly conductive cationic state was already observed in VDAC1 extracted from *Neurospora crassa* or rat liver mitochondria 32, such a difference between native and recombinant VDAC1 from yeast or human has not been detected so far; rather many papers agree with the fact that both samples behave similarly upon PLB analysis in terms of conductance, voltage dependence and selectivity. Conversely, specific functional differences were noticed in the case of human VDAC3. The first electrophysiological characterization of VDAC3 was obtained by heterologous expression in bacteria, which produced proteins able to form pores in artificial membranes with a very low conductance (100 pS in 1 m KCl, channels about 35–40 times smaller than both VDAC1 and VDAC2) 20. However, the work by Okazaki *et al*. showed that the recombinant VDAC3 could form channels with conductance similar to the other isoforms 33. In this work, although the proteins were obtained with very similar protocols, the presence of reducing agents in preparation buffers and in PLB experiments led to the formation of channels of normal conductance. Moreover, channels of regular conductance were also obtained by using human VDAC3 directly extracted in the same conditions from yeast mitochondria 27. At variance was that VDAC3 purified in the absence of reducing agents showed different electrophysiological features 21. The reason for such differences was later attributed to the presence of specific post‐translational modifications (PTMs), in particular to irreversible oxidation states of cysteine residues, as demonstrated by electrophysiological characterization in 21 and recently confirmed by high‐resolution mass spectrometry 34, 35.

We thus hypothesize that also the differences between native and recombinant yVDAC2 could be explained by naturally occurring PTMs. While the native protein is isolated directly from yeast mitochondria under native conditions, the recombinant protein is produced in a heterologous system lacking any eukaryotic PTMs. Beyond oxidation, VDAC proteins undergo phosphorylation of specific amino acid in both physiological and pathological conditions 36, 37, which could be responsible for modulation of voltage sensitivity, as well as other electrophysiological parameters, as has been recently proposed 38, 39. Furthermore, it is known that modifications of amino acid residues change VDAC features. For instance, the treatment of VDAC1 with succinic anhydride abolishes the voltage dependence and changes its selectivity from an anionic to a cationic one 40.

In conclusion, the slight functional differences found in the specific case of *S. cerevisiae* VDAC2 between the native and recombinant proteins do not affect the biological significance of our findings, indicating that yVDAC2 is definitely another member of the VDAC family, being able to form voltage‐dependent channel of about 3.6 nS conductance under our experimental conditions. The behavior of the recombinant protein supports our hypothesis of the presence of specific PTMs in the native one. As a further, intriguing consequence, our results indicate that such differences could be associated to the still unknown role of yVDAC2. Its encoding gene is under‐expressed in normal conditions. It is not the first time, indeed, that an under‐expressed isoform of VDAC was noticed, as was recently found in *Drosophila melanogaster*: in that case, a regulatory function has been associated to the alternative isoform 41.

## Conflict of interest

The authors declare no conflict of interest.

## Author contributions

A. Magrì, AK, and SR performed electrophysiological experiments. A. Magrì, MCDR, and SCN performed protein purification. A. Magrì, A. Messina, HK, and VDP analyzed the data and wrote the manuscript.

## References

[1] Benz R (1994) Permeation of hydrophilic solutes through mitochondrial outer membranes: review on mitochondrial porins. Biochim Biophys Acta 1197, 167–196. 8031826 10.1016/0304-4157(94)90004-3

[2] Colombini M (2004) VDAC: the channel at the interface between mitochondria and the cytosol. Mol Cell Biochem 256–257, 107–115. 10.1023/b:mcbi.0000009862.17396.8d 14977174

[3] Shoshan‐Barmatz V , De Pinto V , Zweckstetter M , Raviv Z , Keinan N and Arbel N (2010) VDAC, a multi‐functional mitochondrial protein regulating cell life and death. Mol Asp Med 31, 227–285. 10.1016/j.mam.2010.03.002 20346371

[4] Messina A , Reina S , Guarino F and De Pinto V (2012) VDAC isoforms in mammals. Biochim Biophys Acta 1818, 1466–1476. 22020053 10.1016/j.bbamem.2011.10.005

[5] Granville DJ & Gottlieb RA (2003) The mitochondrial voltage‐dependent anion channel (VDAC) as a therapeutic target for initiating cell death. Curr Med Chem 16, 1527–1533. 10.2174/0929867033457214 12871124

[6] Shoshan‐Barmatz V , Zakar M , Rosenthal K & Abu‐Hamad S (2009) Key regions of VDAC1 functioning in apoptosis induction and regulation by hexokinase. Biochim Biophys Acta 1787, 421–430. 19094960 10.1016/j.bbabio.2008.11.009

[7] Magrì A , Reina S and De Pinto V (2018) VDAC1 as pharmacological target in cancer and neurodegeneration: focus on its role in apoptosis. Front Chem 6, 108. 29682501 10.3389/fchem.2018.00108 PMC5897536

[8] Cheng EH , Sheiko TV , Fisher JK , Craigen WJ and Korsmeyer SJ (2003) VDAC2 inhibits BAK activation and mitochondrial apoptosis. Science 301, 513–517. 12881569 10.1126/science.1083995

[9] De Pinto V , Reina S , Gupta A , Messina A and Mahalakshmi R (2016) Role of cysteines in mammalian VDAC isoforms’ function. Biochim Biophys Acta 1857, 1219–1227. 26947058 10.1016/j.bbabio.2016.02.020 PMC7115947

[10] Reina S , Guarino F , Magrì A and De Pinto V (2016) VDAC3 as a potential marker of mitochondrial status is involved in cancer and pathology. Front Oncol 6, 264. 28066720 10.3389/fonc.2016.00264 PMC5179545

[11] Hiller S , Graces RG , Malia TJ , Orekhov VY , Colombini M and Wagner G (2008) Solution structure of the integral human membrane protein VDAC‐1 in detergent micelles. Science 321, 1206–1210. 18755977 10.1126/science.1161302 PMC2579273

[12] Bayrhuber M , Menis T , Habeck M , Becker S , Giller K , Villinger S , Vonrhein C , Griesinger C , Zweckstetter M and Zeth K (2008) Structure of the human voltage‐dependent anion channel. Proc Natl Acad Sci USA 105, 15370–15375. 18832158 10.1073/pnas.0808115105 PMC2557026

[13] Ujwal R , Cascio D , Colletier JP , Fahama S , Zhanga J , Torod L , Pinga P and Abramson J (2008) The crystal structure of mouse VDAC1 at 2.3 Å resolution reveals mechanistic insights into metabolite gating. Proc Natl Acad Sci USA 105, 17742–17747. 18988731 10.1073/pnas.0809634105 PMC2584669

[14] Schredelseker J , Paz A , López CJ , Altenbach C , Leung CS , Drexler MK , Chen JN , Hubbell WL and Abramson J (2014) High resolution structure and double electron‐electron resonance of the zebrafish voltage‐dependent anion channel 2 reveal an oligomeric population. J Biol Chem 289, 12566–12577. 24627492 10.1074/jbc.M113.497438 PMC4007448

[15] De Pinto V , Guarino F , Guarnera A , Messina A , Reina S , Tomasello MF , Palermo V and Mazzoni C (2010) Characterization of human VDAC isoforms: a peculiar function for VDAC3? Biochim Biophys Acta 1797, 1268–1275. 20138821 10.1016/j.bbabio.2010.01.031

[16] Blachly‐Dyson E , Song J , Wolfgang WJ , Colombini M and Forte M (1997) Multicopy suppressors of phenotypes resulting from the absence of yeast VDAC encode a VDAC‐like protein. Mol Cell Biol 17, 5727–5738. 9315631 10.1128/mcb.17.10.5727 PMC232421

[17] Magrì A , Di Rosa MC , Tomasello MF , Guarino F , Reina S , Messina A and De Pinto V (2016) Overexpression of human SOD1 in VDAC1‐less yeast restores mitochondrial functionality modulating beta‐barrel outer membrane protein genes. Biochim Biophys Acta 1857, 789–798. 26947057 10.1016/j.bbabio.2016.03.003

[18] Guardiani C , Magrì A , Karachitos A , Di Rosa MC , Reina S , Bodrenko I , Messina A , Kmita H , Ceccarelli M and De Pinto V (2018) yVDAC2, the second mitochondrial porin isoform of *Saccharomyces cerevisiae* . Biochim Biophys Acta 1859, 270–279. 10.1016/j.bbabio.2018.01.008 29408701

[19] Reina S , Magrì A , Lolicato M , Guarino F , Impellizzari A , Maier E , Benz R , Ceccarelli M , De Pinto V and Messina A (2013) Deletion of β‐strands 9 and 10 converts VDAC1 voltage‐dependence in an asymmetrical process. Biochim Biophys Acta 1827, 793–805. 23541892 10.1016/j.bbabio.2013.03.007

[20] Checchetto V , Reina S , Magrì A , Szabò I and De Pinto V (2014) Recombinant human voltage dependent anion selective channel isoform 3 (hVDAC3) forms pores with a very small conductance. Cell Physiol Biochem 34, 842–853. 25171321 10.1159/000363047

[21] Reina S , Checchetto V , Saletti R , Gupta A , Chaturvedi D , Guardiani C , Guarino F , Scorciapino MA , Magrì A , Foti S *et al* (2016) VDAC3 as a sensor of oxidative state of the intermembrane space of mitochondria: the putative role of cysteine residue modifications. Oncotarget 7, 2249–2268. 26760765 10.18632/oncotarget.6850 PMC4823033

[22] Magrì A , Belfiore R , Reina S , Tomasello MF , Di Rosa MC , Guarino F , Leggio L , De Pinto V and Messina A (2016) Hexokinase I N‐terminal based peptide prevents the VDAC1‐SOD1 G93A interaction and re‐establishes ALS cell viability. Sci Rep 6, 34802. 27721436 10.1038/srep34802 PMC5056396

[23] Krammer EM , Saidani H , Prévost M and Homblè F (2014) Origin of ion selectivity in *Phaseolus coccineus* mitochondrial VDAC. Mitochondrion 19, 206–213. 24742372 10.1016/j.mito.2014.04.003

[24] Colombini M (1980) Structure and mode of action of a voltage dependent anion‐selective channel (VDAC) located in the outer mitochondrial membrane. Ann N Y Acad Sci 341, 552–563. 6249159 10.1111/j.1749-6632.1980.tb47198.x

[25] Benz R , Schmid A and Dihanich M (1989) Pores from mitochondrial outer membranes of yeast and a porin‐deficient yeast mutant: a comparison. J Bioenerg Biomembr 21, 439–450. 2478530 10.1007/BF00762516

[26] De Pinto V , Benz R , Caggese C and Palmieri F (1989) Characterization of the mitochondrial porin from *Drosophila melanogaster* . Biochim Biophys Acta 987, 1–7. 2480813 10.1016/0005-2736(89)90447-1

[27] Karachitos A , Grobys D , Antoniewicz M , Jedut S , Jordan J and Kmita H (2016) Human VDAC isoforms differ in their capability to interact with minocycline and to contribute to its cytoprotective activity. Mitochondrion 28, 38–48. 26994639 10.1016/j.mito.2016.03.004

[28] Schein SJ , Colombini M and Finkelstein A (1976) Reconstitution in planar lipid bilayers of a voltage‐dependent anion‐selective channel obtained from paramecium mitochondria. J Membr Biol 30 (2), 99–120. 1011248 10.1007/BF01869662

[29] Colombini M (2016) The VDAC channel: molecular basis for selectivity. Biochim Biophys Acta 1863 (10), 2498–2502. 26826035 10.1016/j.bbamcr.2016.01.019

[30] Magrì A and Messina A (2017) Interactions of VDAC with proteins involved in neurodegenerative aggregation: an opportunity for advancement on therapeutic molecules. Curr Med Chem 24 (40), 4470–4487. 28571556 10.2174/0929867324666170601073920

[31] Hiller S and Wagner G (2009) The role of solution NMR in the structure determinations of VDAC‐1 and other membrane proteins. Curr Opin Struct Biol 19 (4), 396–401. 19665886 10.1016/j.sbi.2009.07.013 PMC2739811

[32] Pavlov E , Grigoriev SM , Dejean LM , Zweihorn CL , Mannella CA and Kinnally KW (2005) The mitochondrial channel VDAC has a cation‐selective open state. Biochim Biophys Acta 1710, 96–102. 16293222 10.1016/j.bbabio.2005.09.006

[33] Okazaki M , Kurabayashi K , Asanuma M , Saito Y , Dodo K and Sodeoka M (2015) VDAC3 gating is activated by suppression of disulfide‐bond formation between the N‐terminal region and the bottom of the pore. Biochim Biophys Acta 1848, 3188–3196. 26407725 10.1016/j.bbamem.2015.09.017

[34] Saletti R , Reina S , Pittalà MG , Belfiore R , Cunsolo V , Messina A , De Pinto V and Foti S (2017) High resolution mass spectrometry characterization of the oxidation pattern of methionine and cysteine residues in rat liver mitochondria voltage‐dependent anion selective channel 3 (VDAC3). Biochim Biophys Acta 1859, 301–311. 10.1016/j.bbamem.2016.12.003 27989743

[35] \Saletti R , Reina S , Pittalà MGG , Magrì A , Cunsolo V , Foti S and De Pinto V (2018) Post‐translational modification of VDAC1 and VDAC2 cysteines from rat liver mitochondrial. Biochim Biophys Acta 1859, 806–816. 10.1016/j.bbabio.2018.06.007 29890122

[36] Kerner J , Lee K , Tandler B and Hoppel CL (2012) VDAC proteomics: post‐translation modifications. Biochim Biophys Acta 1818, 1520–1525. 22120575 10.1016/j.bbamem.2011.11.013 PMC4120668

[37] Lee H , Lara P , Ostuni A , Presto J , Johansson J , Nilsson I and Kim H (2014) Live‐cell topology assessment of URG7, MRP6_102_ and SP‐C using glycosylatable green fluorescent protein in mammalian cells. Biochem Biophys Res Commun 450 (4), 1587–1592. 25034329 10.1016/j.bbrc.2014.07.046

[38] Gupta R and Ghosh S (2017) Phosphorylation of purified mitochondrial Voltage‐Dependent Anion Channel by c‐Jun N‐terminal Kinase‐3 modifies channel voltage‐dependence. Biochim Open 4, 78–87. 29450145 10.1016/j.biopen.2017.03.002 PMC5802065

[39] Miglionico R , Gerbino A , Ostuni A , Armentano MF , Monné M , Carmosino M and Bisaccia F (2016) New insights into the roles of the N‐terminal region of the ABCC6 transporter. J Bioenerg Biomembr 48 (3), 259–267. 26942607 10.1007/s10863-016-9654-z

[40] Doring D and Colombini M (1985) Voltage dependence and ion selectivity of the mitochondrial channel, VDAC, are modified by succinic anhydride. J Membrain Biol 83 (1–2), 81–86. 10.1007/BF01868740 2582125

[41] Leggio L , Guarino F , Magrì A , Accardi‐Gheit R , Reina S , Specchia V , Damiano F , Tomasello MF , Tommasino M and Messina A (2018) Mechanism of translation control of the alternative *Drosophila melanogaster* Voltage Dependent Anion Channel 1 mRNAs. Sci Rep 8, 5347. 29593233 10.1038/s41598-018-23730-7 PMC5871876

